# Growth and fecundity of fertile *Miscanthus *× *giganteus* (“PowerCane”) compared to feral and ornamental *Miscanthus sinensis* in a common garden experiment: Implications for invasion

**DOI:** 10.1002/ece3.3134

**Published:** 2017-06-15

**Authors:** Maria N. Miriti, Tahir Ibrahim, Destiny Palik, Catherine Bonin, Emily Heaton, Evans Mutegi, Allison A. Snow

**Affiliations:** ^1^ Department of Evolution, Ecology and Organismal Biology The Ohio State University Columbus OH USA; ^2^ Department of Agronomy Iowa State University Ames IA USA

**Keywords:** biofuel feedstock, *Bromus inermis*, *Panicum virgatum*, “PowerCane”, risk assessment

## Abstract

Perennial grasses are promising candidates for bioenergy crops, but species that can escape cultivation and establish self‐sustaining naturalized populations (feral) may have the potential to become invasive. Fertile *Miscanthus *× *giganteus*, known as “PowerCane,” is a new potential biofuel crop. Its parent species are ornamental, non‐native *Miscanthus* species that establish feral populations and are sometimes invasive in the USA. As a first step toward assessing the potential for “PowerCane” to become invasive, we documented its growth and fecundity relative to one of its parent species (*Miscanthus sinensis*) in competition with native and invasive grasses in common garden experiments located in Columbus, Ohio and Ames, Iowa, within the targeted range of biofuel cultivation. We conducted a 2‐year experiment to compare growth and reproduction among three *Miscanthus* biotypes—”PowerCane,” ornamental *M. sinensis*, and feral *M. sinensis*—at two locations. Single *Miscanthus* plants were subjected to competition with a native grass (*Panicum virgatum*), a weedy grass (*Bromus inermis*), or no competition. Response variables were aboveground biomass, number of shoots, basal area, and seed set. In Iowa, all *Miscanthus* plants died after the first winter, which was unusually cold, so no further results are reported from the Iowa site. In Ohio, we found significant differences among biotypes in growth and fecundity, as well as significant effects of competition. Interactions between these treatments were not significant. “PowerCane” performed as well or better than ornamental or feral *M. sinensis* in vegetative traits, but had much lower seed production, perhaps due to pollen limitation. In general, ornamental *M. sinensis* performed somewhat better than feral *M. sinensis*. Our findings suggest that feral populations of “PowerCane” could become established adjacent to biofuel production areas. Fertile *Miscanthus *× *giganteus* should be studied further to assess its potential to spread via seed production in large, sexually compatible populations.

## 1 INTRODUCTION

Perennial, non‐food plants that are bred to achieve higher growth rates with minimal chemical inputs are prime candidates for biofuel development (Somerville, Youngs, Taylor, Davis, & Long, 2010; Tilman, Hill, & Lehman, 2006). These feedstock candidates may also possess stress‐tolerant traits such as high nutrient‐ and water‐use efficiencies, allowing them to grow on marginal land (Quinn, Allen, & Stewart, 2010; Smith & Barney, 2014). An ongoing ecological concern, however, is that these traits may promote weediness of biofuel cultivars, causing unintended disturbance within neighboring natural areas (Clark et al., 2014; Owens et al., 2013; Somerville et al., 2010). Presumed economic and ecological benefits of new bioenergy crops must be balanced against environmental risk (Raghu, Spencer, Davis, & Wiedenmann, 2011; Raghu et al., 2006). Research on possible risks is ongoing (reviewed in Barney, 2014) with the aim of minimizing unintended consequences of biofuel development such as promoting species’ invasions.

Grass species in the Asian genus *Miscanthus* present both opportunities and challenges for biofuel cultivation. Due to high rates of biomass accumulation, cold tolerance, and stable performance across broad climate gradients, *Miscanthus sinensis*,* Miscanthus sacchariflorus*, and *M. *× *giganteus* (a hybrid between tetraploid *M. sacchariflorus* and diploid *M. sinensis*) are promising candidates for extensive biofuel production in North America, Europe, and China (Arundale et al., 2015; Friessen, Peixoto, Busch, Johnson, & Sage, 2014; Heaton, Clifton‐Brown, Voigt, Jones, & Long, 2004). The hybrid *M.  *× *giganteus* can be sterile (triploid) or fertile (e.g., tetraploid) (Sacks, Jakob, & Gutterson, 2013; Sacks, Juvik, Lin, Stewart, & Yamada, 2013). A sterile triploid hybrid of *M. *× *giganteus* has been tested as a biofuel in Europe and the USA and produces greater amounts of annual biomass compared to its parent species and other potential perennial grass biofuel candidates (Heaton, Dohleman, & Long, 2008). In this study, we focus on seed‐producing *M. *× *giganteus,* described below.

*Miscanthus* spp. have been cultivated in North America for over a 100 years (Meyer, Paul, & Anderson, 2010; Schnitzler & Essl, 2015). *Miscanthus sinensis*, a warm‐season, C_4_ perennial bunchgrass, was first used for forage, shelter, and clothing (Chou, 2009). Currently, ornamental varieties of *M. sinensis* are widely planted in the USA and elsewhere (Quinn et al., 2010). *Miscanthus sinensis* can reach heights greater than 3 m and produces large, showy, fan‐shaped panicles with abundant, wind‐dispersed seeds. In the USA, naturalized populations arising from escaped seeds or rhizomes of ornamental *Miscanthus*, hereafter referred to as feral populations, have colonized disturbed areas in a diversity of landscapes (Barney & DiTomaso, 2008; Bonin, Heaton, & Barb, 2014; Hager, Sinasac, Gedalof, & Newman, 2014; Quinn, Matlaga, Stewart, & Davis, 2011; Quinn et al., 2010). Feral *M. sinensis* is documented primarily in the eastern USA (Quinn et al., 2010; Schnitzler & Essl, 2015), while the distribution of feral *M. sacchariflorus* extends further north and west (Bonin et al., 2014; Schnitzler & Essl, 2015). The sterile triploid hybrid, *M. *× *giganteus*, has rarely escaped cultivation (Hager, Rupert, Quinn, & Newman, 2015), but large‐scale plantings of this cultivar are fewer and more recent than those of ornamental cultivars. Less is known about the invasive potential of the fertile hybrid *M. *× *giganteus* (“PowerCane” ^™^ Sacks, Jakob, et al., 2013; Sacks, Juvik, et al., 2013).

Public and private entities are breeding fertile *Miscanthus spp*. (2x or 4x) as germplasm for future biofuel cultivars (Clifton‐Brown et al., 2017). Companies such as Ceres, Inc. (Newbury Park, CA, USA), and Mendel Bioenergy Seeds (Hayward CA, USA) developed fertile *Miscanthus* varieties that may be economically more feasible than vegetatively propagated clones. Mendel Bioenergy Seeds (now owned by Repreve Renewables, Greensboro, NC, USA) has conducted field experiments on a nontransgenic variety of *M. *× *giganteus* called “PowerCane” (Sacks, Juvik, et al., 2013). Although breeding efforts have ceased for *M. *× *giganteus “*PowerCane,” research directed to improve fertile hybrids of *Miscanthus* for biofuel production are ongoing, with major progress in the European Union (Clifton‐Brown et al., 2017).

Quantitative ecological studies are needed to investigate the potential of biofuel feedstock biotypes such as fertile *M*.* *× *giganteus* to escape cultivation and establish invasive populations (Barney, 2014). By identifying the conditions that favor germination, survival and growth, these studies complement screening assessments that are designed to prevent or mitigate unintended invasions of candidate biofuel species (Flory, Lorentz, Gordon, & Sollenberger, 2012). Mechanisms for establishment of feral populations of *Miscanthus* are species dependent, with *M. sacchariflorus* spreading largely from rhizomes rather than seeds (Bonin et al., 2014; Hager, Rupert, et al., 2015; Mutegi et al., 2016). In contrast, seed dispersal is the primary mechanism for population growth in *M. sinensis* (e.g., Quinn et al., 2010, 2011). Triploid *M. *× *giganteus* is sterile and therefore less likely to establish feral populations than other *Miscanthus* biotypes; this cultivar is propagated vegetatively, largely from rhizomes (Heaton et al., 2010). The fertile, seed‐producing tetraploid, “PowerCane,” has been developed to reduce growers’ propagation costs associated with planting rhizomes. Smith and Barney (2014) reported high seedling mortality and low emergence for “PowerCane,” which could suggest a low risk of escape from cultivation. However, we conducted seed addition experiments in Iowa and Ohio and found that “PowerCane” had higher establishment and produced more biomass per plot than ornamental or feral *M. sinensis* at both locations (Bonin et al., 2017). Considering this ability of “PowerCane” to escape cultivation, it is important to assess its performance relative to neighboring vegetation.

Once a feral population is established, its persistence and invasiveness are regulated by interactions with neighboring vegetation (Flory et al., 2012; Hager, Quinn, Barney, Voigt, & Newman, 2015). Perennial species that are strong competitors may possess a stronger ability to persist and spread compared to those that show reduced growth in the presence of competitors. To further assess the potential for “PowerCane” to persist and become invasive outside of cultivation, we used common garden experiments to examine responses of “PowerCane” to three competition treatments and compared these responses to those of ornamental and feral *M. sinensis* (below, we refer to these three taxa as biotypes). Experimental plots were located at two sites within the targeted range of cultivation for *Miscanthus*: in central Ohio and central Iowa. Response variables of the three biotypes were biomass, basal area, number of shoots, and number of seeds per plant, measured 2 years after planting. The first three traits were used to characterize vegetative growth responses, and seed number was used to assess reproductive output. We used contrast analysis to evaluate if the relative performance of “PowerCane” (compared to ornamental and feral *M. sinensis*) was altered by competition treatments, and to assess the presence of a statistical interaction between biotype and competition.

## 2 METHODS

### 2.1 Study sites

**Figure 1:**
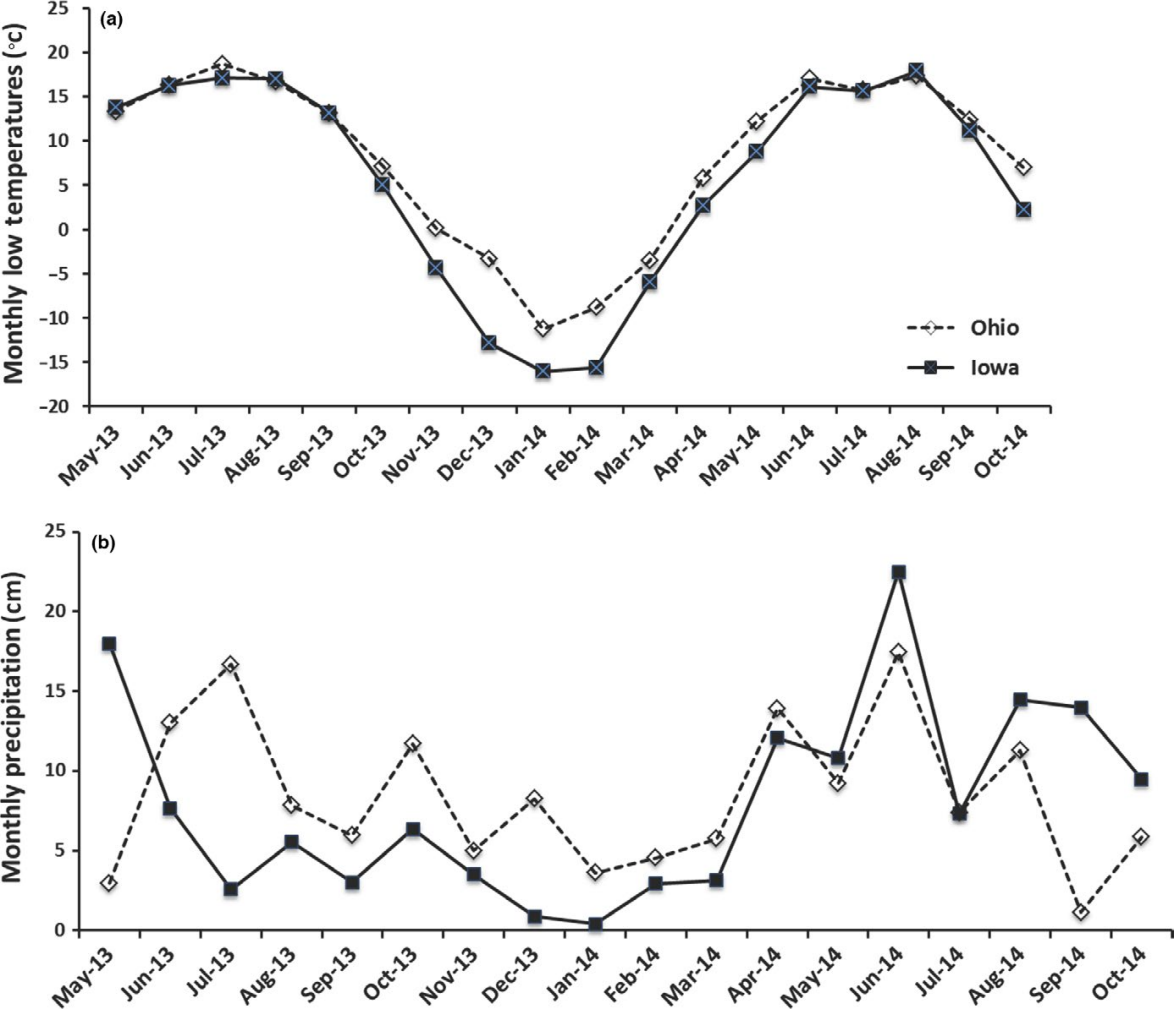
Weather data for Boone County (Iowa) and Franklin County (Ohio) for the (a) monthly low temperatures, and (b) monthly total precipitation during the study period

The two study sites were located at The Ohio State University—Waterman Farm in Columbus, Ohio (40.0079**°**N, 83.0359**°**W), and at Iowa State University Sorenson Farm near Ames, Iowa (42.0300°N, 93.8000°W). Local monthly average rainfall and minimum–maximum temperatures during the growing season (May–October) were obtained from Ohio Agricultural Research and Development Center and the Iowa Environmental Mesonet (Figure 1).

### 2.2 *Miscanthus* biotypes

We examined three *Miscanthus* biotypes: “PowerCane,” ornamental *M. sinensis* (“Jelitto”), and feral *M. sinensis* (Table 1). Seeds for *M. *× *giganteus “*PowerCane” were donated by Mendel Bioenergy Seeds, and ornamental seeds were purchased from Jelitto Perennial Seeds (cultivar No. ZA274). For feral *M. sinensis*, seeds were collected from populations in Dallison, West Virginia; Williamstown, West Virginia; and Marietta, Ohio. We were not able to include *M. sacchariflorus*, the other parent species of “PowerCane,” because feral populations rarely set seed (Mutegi et al., 2016) and commercial cultivars are propagated vegetatively.

**Table 1:** Description of *Miscanthus* biotypes used in this experiment, their classification, origin, and GPS coordinates

Biotype	Ploidy	Classification	Origin	GPS coordinates (latitude/longitude)
*M*.* *× *giganteus “*PowerCane”	4x	Biofuel cultivar	Mendel Biotechnology Seeds	N/A
*M. sinensis* “Jelitto”	2x	Ornamental cultivar	Jelitto Seed Company	N/A
*M. sinensis “*Dallison”	2x	Feral	Dallison, West Virginia	39.25, 81.38
*M. sinensis* “Marietta”	2x	Feral	Marietta, Ohio	39.48, 81.30
*M. sinensis* “Williamstown”	2x	Feral	Williamstown, West Virginia	39.40, 81.44

### 2.3 Competitor species

*Panicum virgatum “*Cave‐in‐Rock” (switchgrass) and *Bromus inermis* (bromegrass) were the competitor species in this experiment. *Panicum virgatum*, a warm‐season C_4_ perennial grass native to North America, is typically found in prairie grasslands. Similar to *M. sinensis, P. virgatum* is being considered for biofuel feedstock. In contrast, *B. inermis* is a cool season C_3_ perennial grass native to Hungary and Russia. *Bromus inermis*, first introduced to North America in the late 1800s as a forage plant, is considered invasive due to its ability to rapidly divide and spread through rhizomes and form sod patches, reaching heights of >1 m. This species establishes in agricultural fields, forests, pastures and has caused drastic ecological alterations by establishing large populations in native prairies (Dillemuth, Rietschier, & Cronin, 2009). These two species have overlapping ranges through most of the continental United States, including our study sites and represent contrasting competitors with which to evaluate the success of escaped *Miscanthus*. Seeds for both competitor species were purchased from Millborn Seeds Inc., SD, USA.

### 2.4 Experimental design

Two common garden experiments were established: one in Ohio and the other in Iowa. Each used a randomized block, factorial design to reduce bias due to underlying habitat heterogeneity. Treatment variables were biotype and competition. The focal *Miscanthus* biotypes were planted in three competition treatments: no competition, competition with *P. virgatum*, or competition with *B. inermis*, with 15 replicates per biotype in each level of competition. Initially, we initially considered each feral population of *M. sinensis* as a separate biotype, with 15 replicates for each population. Later, these were grouped together for data analyses because differences among the feral populations were not significant. At each site, the experimental area was divided into 15 rows, with row representing a single block, and one plot per row was randomly assigned to one of the 15 different treatment x biotype combinations resulting in a total of 225 plots.

**Figure 2:**
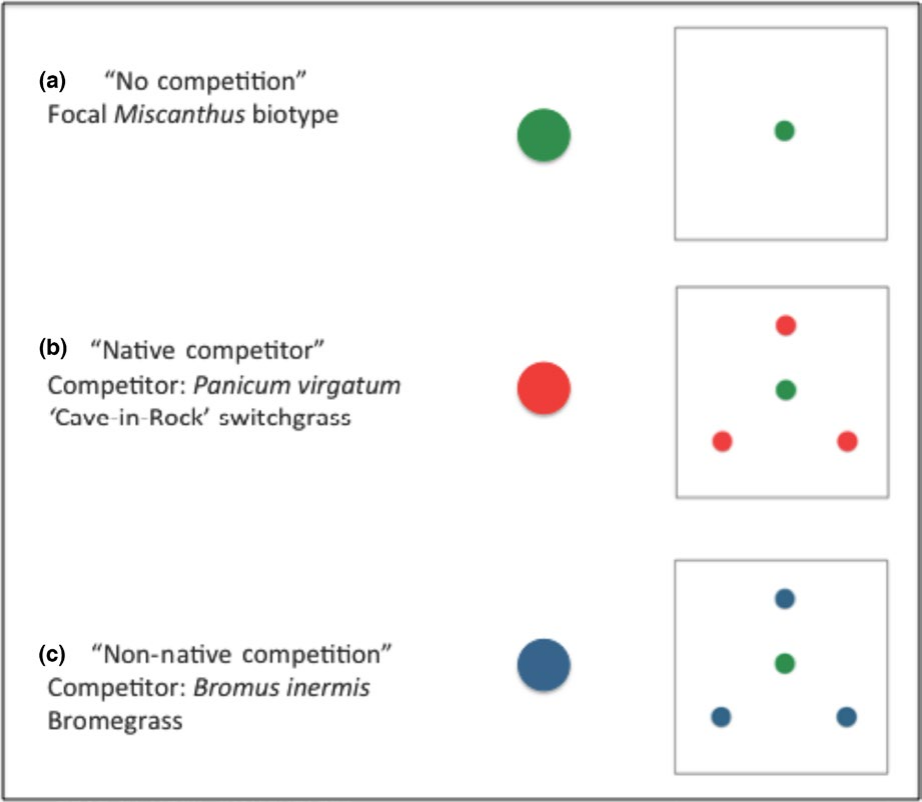
Schematic diagrams of the layouts for experimental plots

An individual plot measured 1.5 m × 1.5 m, with a 1.5‐m buffer zone. Each plot had one focal *Miscanthus* plant at its center. Competition treatments consisted of three individuals of a single competitor species planted 30 cm from the focal plant (Figure 2). Plots with no competition consisted of the focal biotypes at the center of the plot and were used as controls to understand the effect competition from *B. inermus* and *P. virgatum* on *Miscanthus’* productivity (Figure 2).

### 2.5 Planting methods

*Miscanthus* seeds were cleaned in a 5% bleach solution for 5 min to kill fungal pathogens, and were subsequently rinsed twice using distilled water. In Ohio, seeds were germinated in Petri dishes with 1% agar solution and stored inside an incubation chamber at 35°C. In Iowa, seeds were started in germination boxes. Once radicles protruded through their seed coat, the seedlings were transplanted into either 2.54‐cm peat pots with Fafard^®^ 2 Mix (Ohio) or 2.54‐cm plastic pots with Sunshine^®^ LCI Mix (Iowa). In the third week of April 2013, the Ohio seedlings were placed under misting benches (watered automatically three times a day, for 3 min) for 6 weeks at the Ohio State University Biological Sciences Greenhouses. As seeds germinated (from 25 March 2013–15 April 2014) in Iowa, they were transferred to the glasshouse. To ensure initial survival prior to transplanting, plants received liquid fertilizer treatments from Scotts Pro 20‐10‐20 Peat‐Lite Special at 200 ppm (Ohio) or Peters Excel^®^ 15‐5‐15 Cal–Mag Special and supplemented with Miracid (Iowa).

Two weeks prior to planting, each field was sprayed with both 2,4 D and glyphosate to eliminate unwanted competition from weedy species. The same site preparation techniques were implemented in Iowa and Ohio. Then, to eliminate local weeds near the focal plants and their competitors, a weed blocking fabric was used in Iowa and ~15 cm of mulch was spread along the rows in Ohio.

Once all seedlings reached at least 5 cm, they were transplanted into the experimental garden plots. Seedlings were planted between 27–28 May 2013 (Ohio), and 11–13 June 2013 (Iowa). To reduce mortality from transplant shock, plants were watered during the first month if rain was not adequate and plants showed wilting. During the growing season, the rows between experimental plots were weeded every week and sprayed with either 2,4 D or glyphosate, taking care not to injure the experimental plants (no injury was observed). All focal plants were measured and harvested at ground level at the end of the second growing season on 14 October 2014.

### 2.6 Data collection

At the end of the second growing season, we recorded total dry biomass, basal area, total number of shoots (reproductive and nonreproductive), and seed production (estimated based on three panicles per plant). We measured the basal diameter for every focal plant and used it to calculate basal area (Area = ¼π*d* ^2^). The total number of reproductive and nonreproductive shoots were counted and combined to provide the total number of shoots. A subsample (½, ¼, or ⅛, with a larger fraction for smaller plants) of the fresh biomass was collected and weighed for each plot to limit the amount of time spent harvesting. To estimate dry weights, ten plots of each focal biotype were randomly subsampled for fresh biomass (~200 g) and dried at 37.8°C, until the samples reached a constant weight (~2 weeks). The dry weight from each subsample was divided by its original weight to get a fresh to dry weight ratio. The average fresh to dry weight ratio for each focal biotype was used as a conversion factor to convert fresh weight to dry weight, and finally scaled up depending on the fraction that was sampled to get the total dry weight per plot. To estimate seed production, a subsample of three panicles were collected and processed, using rubber blocks and blower techniques to extract seeds. The seeds were then weighed in parcels of three subsamples of 50 seeds. This weight was used to extrapolate the total number of seeds from the three collected panicles. The number of seeds per plant was calculated as the number of flowering panicles x the mean number of seeds per panicle.

### 2.7 Data analysis

A general linear mixed‐effect model was used to measure the effects of biotype, competition, and their interaction on biomass, basal area, number of shoots, and estimated seed production. Block was included as a random factor. With the exception of number of shoots, variables were log‐transformed to meet homoscedasticity requirements. Tukey's HSD contrasts were used to determine significant differences within treatments. All analyses were completed using JMP (v12, SAS Institute Inc., Cary, NC, 1989–2007). As noted above, because there were no significant differences in the responses among feral populations, these were combined into a single category.

## 3 RESULTS

During the first winter of the experiment, heavy mortality occurred in Iowa, most likely due to colder and drier weather conditions (National Climatic Data Center Oct–Dec 2013, Figure 1). As a result, we present analyses of the Ohio site only.

**Figure 3:**
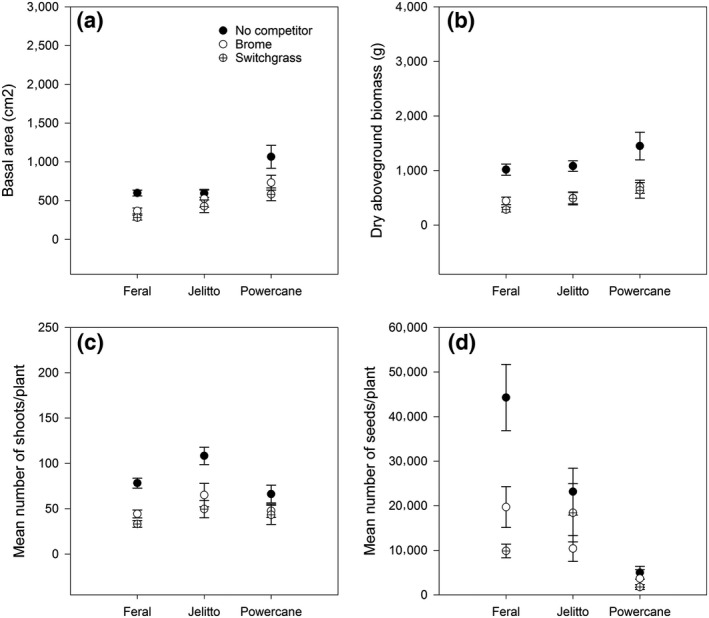
The performance of each *Miscanthus* biotype at each competition treatment (mean ± standard error). The legend for all symbols is located in the upper right corner of the Basal area figure (a). All biotypes performed better in the absence of competition (closed circles). Although there was no significant interaction between treatments, Jelitto showed more variable responses to competitors than feral plants or “PowerCane.” In contrast, seed production for feral plants was more strongly suppressed by competition than for Jelitto or “PowerCane”

**Figure 4:**
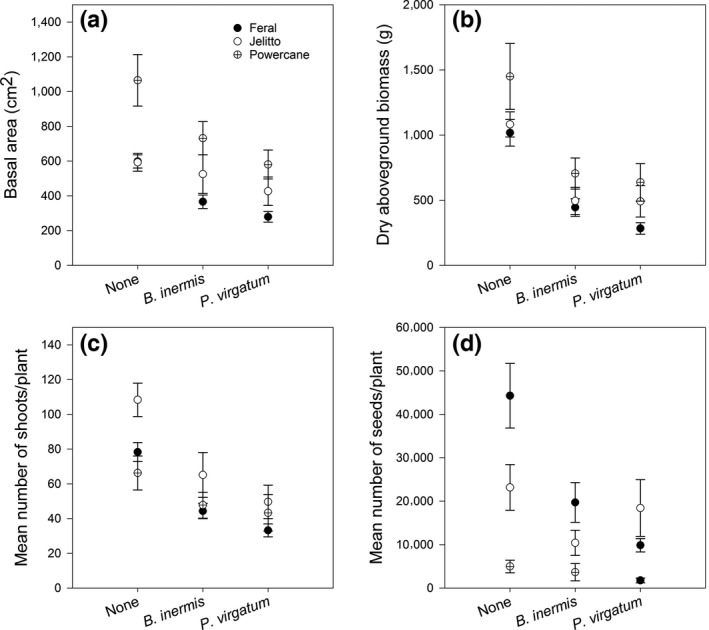
Competitive responses for each *Miscanthus* biotype for (a) basal area, (b) aboveground biomass, (c) number of shoots/plant, and (d) number of seeds/plant. There were no significant interactions among the main effects of biotype and competition, but there are notable differences among biotypes in the strength of the response to competition for each response variable. For all biotypes, competition reduced plant performance

For all dependent variables, the effects of competition and biotype were significant and there were no significant interactions between treatments (Table 2). Thus, the effects of competition were similar within biotypes (Figure 3). Competition from *P. virgatum* vs. competition from *B. inermis* generally the growth of *Miscanthus*; although not significant, the intensity of this inhibition differed among the biotypes (Figure 4). Across competition treatments, “PowerCane” had greater biomass and basal area than ornamental or feral *Miscanthus*, but its seed production was an order of magnitude lower relative to the other biotypes (Figure 4). Results are summarized below.

**Table 2:** Summary of ANOVAs for the effects of biotype (three levels), competition treatment (three levels), and their interaction for each measured variable in the Ohio common garden experiment

Variables	Source	Biotype	Competition	Biotype × competition
Basal area (cm^2^)	F‐Stat	19.69	15.21	0.91
	*p*‐Value	<.0001	<.0001	.46
Dry weight (g)	F‐Stat	8.03	22.81	0.73
	*p*‐Value	<.001	<.0001	.57
Number of shoots	F‐Stat	7.54	23.45	1.09
	*p*‐Value	<.001	<.0001	.36
Seed production	F‐Stat	19.10	3.24	0.29
	*p*‐Value	<.0001	<.05	.88

**Figure 5:**
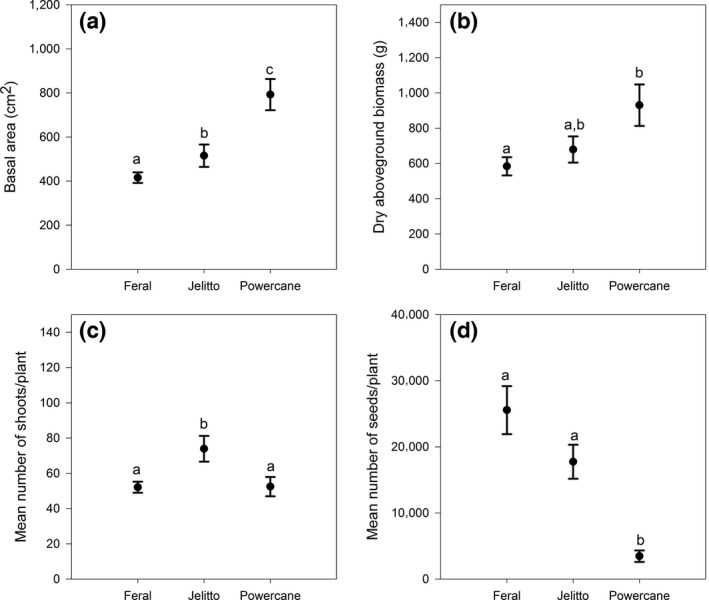
The performance of each *Miscanthus* focal biotypes averaged across all competition treatments (mean ± standard error of treatments per biotype) for (a) basal area, (b) aboveground biomass, (c) number of shoots/plant, and (d) seeds/plant. Feral plants are combined data from three locations (*N* = 122). Jelitto is an ornamental cultivar (*N* = 41), and “PowerCane” a potential biofuel cultivar (*N* = 42). Lowercase letters represent significant differences using Tukey's HSD Test

**Figure 6:**
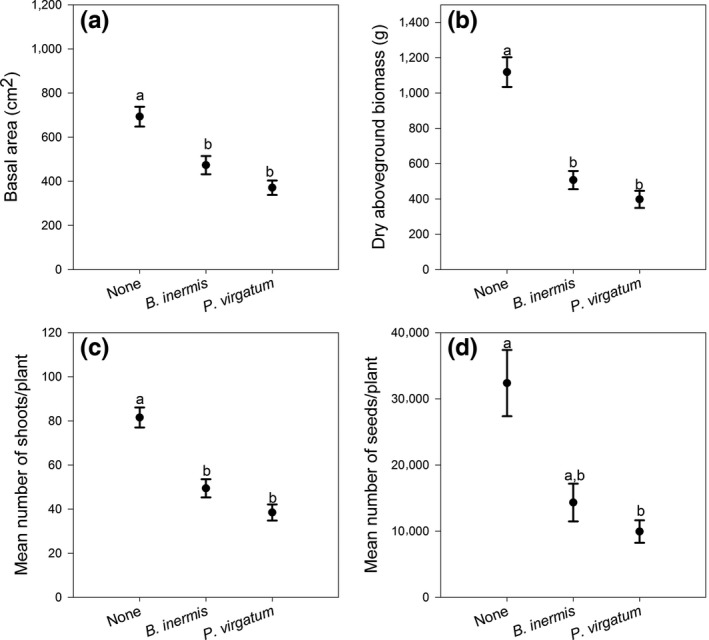
Competitive responses averaged across all *Miscanthus* biotypes to competition for (a) basal area, (b) aboveground biomass, (c) number of shoots/plant, and (d) seeds/plant. None refers to no competitor present (*N* = 68), Brome represents competition with *Bromus inermis* (*N* = 70), and Switchgrass refers to competition with *Panicum virgatum* (*N* = 67). For all plots, means ± standard error are depicted. Lowercase letters represent significant differences using Tukey's HSD

“PowerCane” plants had a larger basal area than those from “Jelitto,” which was in turn larger than the feral plants (Figure 5, Tukey's HSD α = 0.05). Dry biomass was also dependent on biotype (*p* = .0005) with “PowerCane” producing more dry biomass than the feral plants (Tukey's HSD α = 0.05, Figure 5). Jelitto plants produced more shoots than “PowerCane” and feral plants (Tukey's HSD α = 0.05, Figure 5), and plants grown without competition had greater numbers than those grown with competition (Figure 6). Plants grown in competition with *P. virgatum* produced fewer seeds than those without competition, but not for those in competition with *B. inermis* (Figure 6, Tukey's HSD α = 0.05). Among the biotypes, feral and *M. sinensis “*Jelitto” plants produced significantly more seeds than “PowerCane” (Figure 5, Tukey's HSD α = 0.05).

## 4 DISCUSSION

All *Miscanthus* biotypes suffered a reduction in biomass, basal area, total number of shoots, and seed production in response to competition, as expected. Our results show that competition did not modify the effects of biotype; therefore, our discussion focuses on the overall differences among biotypes. A major conclusion from our study is that “PowerCane” generally performed as well or better than ornamental or feral biotypes in terms of vegetative growth, but its seed production was an order of magnitude lower than the other biotypes. We discuss this low seed production below. This result suggests that if “PowerCane” establishes feral populations, they should persist at least as well as feral *M. sinensis*.

The relative invasiveness of species introduced for agriculture or horticulture has been associated with factors such as high productivity, disease resistance, flowering phenology, and seed production (Knight, Havens, & Vitt, 2011; Simberloff, 2008). Although “PowerCane” did not produce as many seeds as the other *Miscanthus* biotypes, Knight et al. (2011) concluded that large differences in fecundity may have a relatively small effect on the population growth rates of long‐lived plants that have the potential to be invasive because population growth models of invasive plants, in particular perennial species, showed that population growth rate responded little to reductions in fecundity. The superior growth and potentially large planting area could contribute to an invasion risk of “PowerCane,” in particular if pollen limitation (reviewed in Knight et al., 2005) explains the low seed production in our experiment. “PowerCane” was intended to be grown in managed settings, with annual harvest of inflorescences prior to seed maturation, but the volatile nature of bioenergy markets has made commercial fields a potential liability if abandoned.

Regarding competition, our findings are consistent with those of Barney, Mann, Kyser, and DiTomaso (2012), who documented better performance of *M. *× *giganteus* in noncompetitive lowland sites compared to competitive upland sites in California. Although we did not examine the competitive effect (sensu Goldberg & Fleetwood, 1987) of *Miscanthus*, it has been reported that naturalized *M. sinensis* suppressed *P. virgatum* in a glasshouse setting, suggesting that it may be a stronger competitor than *P. virgatum* in the field (Meyer et al., 2010). There was greater variance in the response of “PowerCane” for basal area and biomass compared to the *M. sinensis* biotypes (Figure 3), but overall, the absence of an interaction between biotype and competition showed that relative differences among the biotypes diminished in the presence of competition. Further, we found that the competitor effects were the same across all biotypes independent of the competitor. Taken together, these results suggest that the greater growth of “PowerCane” may not translate to stronger invasive ability compared to its parent species, *M. sinensis*, at least when feral populations are established in relatively undisturbed, vegetated areas.

Low seed set of “PowerCane” warrants further examination because (1) as the only biotype with a 4x ploidy, “PowerCane” seed set may have been pollen limited, (2) life‐history trade‐offs may contribute to a negative correlation between allocation to biomass and reproduction (Stearns, 1992; Weiner, 2004), and (3) being derived from a half‐sib cross (Sacks, Juvik, et al., 2013) “PowerCane” may suffer from inbreeding depression. If pollen limitation explains low seed set, then extensive plantings of “PowerCane” would be expected to have greater levels of seeds set per plant than observed in our study. On the other hand, because resource limitations to reproduction can respond to local conditions (Obeso, 2004; Pulido et al., 2014), examination of trade‐offs between allocation to growth or reproduction across a broad geographic range is necessary to understand potential propagule pressure of “PowerCane.” Low seed production alone does not eliminate potential invasion of “PowerCane” because Smith and Barney (2014) found that sites with available bare ground and low resident plant competition were invaded by *M. *× *giganteus*. Our parallel seed addition study (Bonin et al., 2017) also suggests that low seed set may not be as limiting to “PowerCane” as it might be for less vigorous *Miscanthus* varieties. Finally, inbreeding depression across generations of “PowerCane” may reduce seed viability or the competitive ability of resulting plants. Because inbreeding depression in outcrossing plants varies widely, empirical studies are needed to assess such consequences for “PowerCane.” Our separate seed addition study further indicates that “PowerCane” established from seed produces large, competitive, flowering culms after 2 years (Bonin et al., 2017). Even if seed viability is low, these plants may become invasive asexually as *M. sacchariflorus*, a congener with low seed set (Mutegi et al., 2016) has already done in the United States (Bonin et al., 2014). The general finding is that once established, “PowerCane” plants persist.

Our ability to examine geographic variation in competitive responses was not possible due to major mortality after the first winter at the Iowa site, which experienced colder temperatures and lower precipitation prior to the second growing season than in Ohio (Figure 1). This result has relevance because dispersal ability and cold tolerance are important controls for initial establishment of *M. sinensis* (e.g., Quinn et al., 2010, 2011) emphasizing the importance of the first year of establishment for long‐term persistence.

Our experiment contributes to the ongoing risk assessment of *Miscanthus* cultivation. International recognition of human‐induced climate change has stimulated an enormous amount of research and development toward producing plant‐based renewable energy to reduce reliance on fossil fuels and emissions of glasshouse gases (e.g., Heaton et al., 2008; Powlson, Riche, & Shield, 2005). This multifaceted research includes evaluation of potential plant species in terms of their economic costs and benefits affecting land use (Heaton et al., 2008; Somerville et al., 2010), yield (Jeżowski, Głowacka, & Kaczmarek, 2011; Kim, Kim, Jeong, Jang, & Chung, 2012; Powlson et al., 2005), and ecological impact (Field, Campbell, & Lobell, 2008; Mack, 2008; Raghu et al., 2006, 2011; Wiens, Fargione, & Hill, 2011). Our findings suggest that development of seeded varieties of *M. *× *giganteus* may lead to feral populations with invasive potential, especially if larger populations produce more seeds than observed in our small‐scale field experiment. Lack of seed production is the critical trait that allows sterile *M. *× *giganteus* to be white‐listed (Quinn, Gordon, Glaser, Lieurance, & Flory, 2015). We have shown that with the exception of seed production, seeded “PowerCane” produces larger plants and shows comparable performance under competition compared to feral accessions. Taken together with our seed addition experiment (Bonin et al., 2017), our results support the potential of seeded *Miscanthus* to establish feral populations. Further research on limitations to seed production and spread of feral populations will be useful for assessing the invasive potential of “PowerCane.”

## CONFLICT OF INTEREST

None declared.
